# A dynamically tunable human serum albumin biosensor based on topological edge states graphene nanozyme

**DOI:** 10.3389/fbioe.2025.1732804

**Published:** 2026-01-07

**Authors:** Ling Chen, Qiaohong Yao, Jie Chen, Yuxiang Peng, Jiao Xu, Qiang Fu

**Affiliations:** 1 The Central Hospital of Xiangtan, The Amiliated Hospital of Hunan University, Xiangtan, China; 2 Institute of Mathematics and Physics, and Hunan Province Key Laboratory of Materials Surface and Interface Science and Technology, Central South University of Forestry and Technology, Changsha, China; 3 School of Information Science and Engineering, Hunan Women’s University, Changsha, China

**Keywords:** graphene, human serum albumin, optical biosensor, optical communication band, topological edge states

## Abstract

This study presents a novel optical biosensor for human serum albumin (HSA) detection utilizing a heterostructure that integrates topological edge states with graphene. The sensor achieves high-sensitivity detection through optical topological modes and enables dynamic system responsiveness via graphene’s tunable conductivity regulated by Fermi level modulation. Numerical results demonstrate that topological edge state excitation induces a sharp reflectance dip (depth >95%) at 195.5 THz in the optical communication band, exhibiting exceptional responsiveness to refractive index variations while maintaining stability against environmental interference through topological protection. Dynamic optimization is realized through electrostatic gating modulation of graphene’s Fermi energy and layer number, with additional sensitivity enhancements achieved via precise control of sensing layer thickness and refractive index. The integration of topological photonics with two-dimensional materials provides a versatile foundation for developing sensing-therapeutic systems that address current challenges in biomedical applications, demonstrating significant potential for integration with nanozyme-based diagnostic and therapeutic nanotechnology. The platform’s exceptional field enhancement and tunability could potentially augment the imaging sensitivity of nanozyme-based contrast agents, while its precise modulation capabilities may improve therapeutic efficiency through optimized catalytic activity. Furthermore, the robust topological protection mechanism offers enhanced stability crucial for clinical translation, addressing key limitations in current nanozyme technology including biocompatibility concerns and inconsistent catalytic performance. This integrated approach opens new possibilities for miniaturized, tunable, and interference-resistant biosensing systems with significant potential for multimodal synergistic applications in clinical diagnostics and environmental monitoring.

## 1 Introduction

Optical biosensors employ advanced optical transduction mechanisms to achieve precise capture and quantitative analysis of biological information. By converting microscopic biological phenomena or weak biosignals into quantifiable optical characteristics, this technology demonstrates core advantages including non-contact operation, label-free detection, and non-destructive testing (Brongersma et al., 2025), coupled with exceptional anti-interference capability (Wen et al., 2024) and high sensitivity (Mostufa et al., 2024). These distinctive features have enabled its significant ap-plications in heavy metal ion analysis (Zhu et al., 2023), pathogenic microorganism screening (Yu et al., 2020), pharmaceutical component detection (Saliya et al., 2025), and biomarker recognition (Sinibaldi, 2021). Breakthroughs in micro-nano fabrication technologies have driven the field toward miniaturization and integration, yielding innovative sensor architectures based on photonic crystals (Jia and Feng, 2023), carbon nanotube arrays (Farrera et al., 2017), micro-ring resonators (Rosa and Roberto, 2018), toroidal dipole resonance (Liu et al., 2023a), and terahertz plasmons (Xing et al., 2021). Meanwhile, the emerging field of nanozyme-based theranostics faces challenges in real-time monitoring and precise regulation of catalytic efficiency, calling for novel sensing platforms with high sensitivity and tunability.

Optical topological edge states (TES), with their robust transport characteristics (Lang et al., 2012; Lu et al., 2016), have revolutionized the interference resistance and detection sensitivity of op-tical sensors (Lin et al., 2023). Through innovative design of special electromagnetic modes such as topological edge state (Su et al., 2022), researchers have achieved high-precision localized field-enhanced detection of minute physicochemical signals including weak refractive index variations and molecular adsorption, demonstrating 1-2 orders of magnitude sensitivity improvement over conventional sensors (Bao et al., 2022). Diverse technological approaches have emerged in biosensing applications: Dual-band reflective optical sensors utilizing GMR topological edge states enable hemoglobin-specific detection (Liu et al., 2022); topological ring resonator architectures significantly enhance refractive index resolution (Liu et al., 2023b); tunable surface plasmonic crystals simultaneously excite terahertz topological edge and corner states, expanding broadband detection capabilities (Wang, 2021).

Based on this, researchers have been attempting to combine new materials with new mechanisms to obtain novel optical biosensors that are structurally simple yet dynamically controllable (Zhao et al., 2020; Dai et al., 2019; Wu et al., 2018). Human serum albumin (HSA) is the most abundant protein in plasma, responsible for maintaining plasma colloid osmotic pressure, transporting nutrients and metabolic waste, and other critical physiological functions that are vital for maintaining homeostasis in the body (Shastri et al., 2024). Detecting the level of HSA can aid in the diagnosis of liver diseases (such as cirrhosis), kidney diseases (such as nephrotic syndrome), and malnutrition, providing key evidence for clinical condition assessment and treatment plan formulation (Kayani et al., 2024). In recent years, graphene has emerged as a revolutionary discovery in materials science due to its unique physical and chemical properties (Geim, 2009; Geim and Novoselov, 2007). Its atomic-level thickness, high specific surface area, excellent electrical conductivity, and mechanical strength have enabled it to demonstrate enormous potential in fields such as optoelectronics, energy, and biomedicine (Phiri and Mohssin, 2024; Mahalakshmi et al., 2025). In biosensing applications, graphene’s core advantages stem from its dynamically controllable dielectric constant and electrical conductivity, as well as the special electromagnetic response elicited by its metal-like proper-ties. These characteristics provide new ideas for designing high-performance biosen-sors (Grigorenko et al., 2012). Traditional surface plasmon resonance (SPR) sensors often rely on gold films, but the introduction of graphene can enhance the local electromagnetic field and optimize the interface interaction, leading to a significant increase in sensitivity (Jacob et al., 2024). In addition, the di-electric properties of graphene can be dynamically controlled through external electric fields, chemical doping, or layer number adjustments, endowing the sensor with environmental adaptability and adding multi-dimensional performance to the sensor’s regulation capabilities (Shahram et al., 2024). Currently, optical biosensors centered around two-dimensional materials have become a frontier in the field, combining high-sensitivity detection with intelligent integration. They are not only a natural product of the cross-integration of materials science and information technology but also provide revolutionary solutions for application scenarios such as precision medicine and environmental monitoring (Sangeetha et al., 2025).

Building on this premise, this paper presents a novel biosensor for the detection of HSA, leveraging a composite structure integrated with TES in graphene. We demonstrate that the high sensitivity achieved within the optical communication band originates from the localized field enhancement characteristics-specifically, abnormal reflection peaks-induced by optical topological states. Additionally, the tunable conductivity of graphene serves as a foundational attribute for designing adjustable sensing characteristics within this architecture. Moreover, the inherent robustness of optical topological states ensures robust anti-interference capabilities, enabling stable multi-dimensional modulation in graphene-based biosensing systems. We posit that this electronically tunable sensor for the detection of HSA, featuring a simple layered-stacking graphene structure operating in the optical communication band, holds substantial promise for transformative applications in the field of biosensors.

## 2 Materials and methods

**FIGURE 1:**
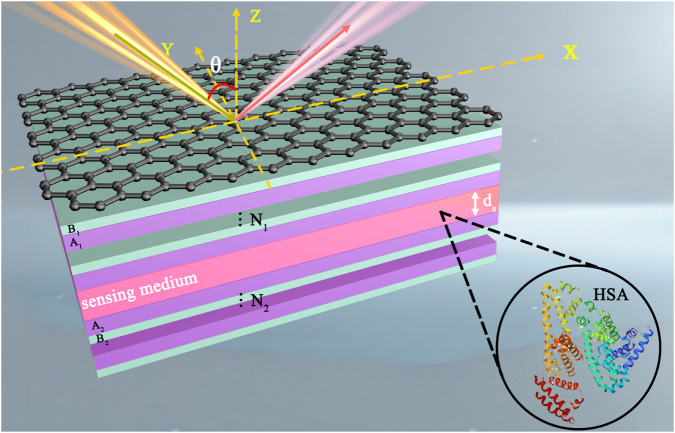
Schematic diagram of a human serum albumin biosensor based on topological edge states heterostructure with graphene.

We propose a heterostructure to excite topological edge states, which is composed of graphene, photonic crystal 1, sensing layer, and photonic crystal 2. Meanwhile, we set up the inlet and outlet of the sensing liquid in the sensing medium layer to make the biosensor model applicable to practical application scenarios, as shown in Figure 1. Graphene is placed at the top of the structure, so the incident light travels through the air and first comes into contact with the graphene at θ . Photonic crystals are composed of two different dielectrics, A and B, whose refractive indexes and thicknesses are respectively expressed as $n_{a},d_{a1},d_{a2},n_{b},d_{b1},d_{b2}$ . The sensing layer is placed between the two photonic crystals and the period of the photonic crystal to “ $N_{1}=N_{2}=4$ ”.

PhC1 and PhC2 are alternately formed by actual optical materials A (SiO2) and B (Si), the refractive indices of dielectrics A and B are assumed to be non-dispersion and set as: $n_{a}=2.82$ , $n_{b}=1.46$ . In addition, the thicknesses of dielectric A and B are set to $d_{a1}=1000\text{ nm}$ , $d_{b1}=680\text{ nm}$ , $d_{a2}=1290\text{ nm}$ and $d_{b2}=600\text{ nm}$ respectively. HSA is often used as the sensing layer of biosensors due to its excellent bio-compatibility, abundant ligand binding sites, and stable physicochemical properties. Here, we choose it as sensing layer with the refractive index $n_{s}=1.365\pm 0.001$ and the thickness of $d_{s}=4000\text{ nm}$ (Malmsten, 1994). As for graphene above the heterostructure, its photoelectric properties are expressed by electrical conductivity considering it’s only one-atom-thick ( 0.34 nm ). Generally speaking, for the terahertz band, only the intra-band conductivity of graphene should be considered (as it far exceeds the inter-band conductivity), but consid-ering that this paper selects the communication band, for the characterization of gra-phene’s conductivity, we choose the sum of in-band and inter-band which can be ap-proximately expressed as:

$$\sigma_{0}=\sigma_{inter}+\sigma_{intra}$$ (1)

$$\sigma_{intra}=\frac{ie^{2}k_{B}T}{\pi h^{2}\left(\omega +\frac{i}{\tau }\right)}\left(\frac{E_{F}}{k_{B}T}+2\ln\left(e^{-\frac{E_{F}}{k_{B}T}}+1\right)\right)$$ (2)

$$\sigma_{inter}=\frac{ie^{2}}{4\pi \hbar }\ln\left|\frac{2E_{F}-\left(\omega +i\tau^{-1}\right)\hbar }{2E_{F}+\left(\omega +i\tau^{-1}\right)\hbar }\right|$$ (3)

where ћ is the simplified Planck constant, e and τ represent the elementary electric charge and the relaxation time, respectively. $E_{F}$ is the Fermi energy and closely related to the carrier density $n_{2D}$ and Fermi velocity of the electron $V_{F}\approx 10^{6}\;m/s$ . In the follow-ing calculations, the Fermi energy and the relaxation time of graphene are taken as $E_{F}=1\text{ eV}$ and τ=1 ps respectively.

In this paper, the traditional and reliable transfer matrix method (Jacob et al., 2024; Shahram et al., 2024) was adopted to evaluate the structural reflection characteristics to reveal the sensing performance. To simplify the analysis, only the TM polarization mode was focused. At this time, the trans-fer relationship at the graphene-dielectric interface can be expressed as:

$$D_{i\to A}=\frac{1}{2}\left[\begin{array}{c} 1+\eta_{\text{iA}}+\xi_{\text{iA}}\\ 1‐\eta_{\text{iA}}+\xi_{\text{iA}} \end{array}\begin{array}{c} 1‐\eta_{\text{iA}}‐\xi_{\text{iA}}\\ 1+\eta_{\text{iA}}‐\xi_{\text{iA}} \end{array}\right],$$ (4)

Where $\eta_{\text{iA}}=\frac{\;\varepsilon_{i}k_{\text{Az}}}{\varepsilon_{A}k_{\text{iz}}}$ and $\xi_{\text{iA}}=\frac{\sigma k_{\text{Az}}}{\varepsilon_{0}\varepsilon_{A}\omega }$ , ${\;k}_{\text{iz}}$ and ${\;k}_{\text{Az}}$ are the wave vector compo-nents of electromagnetic waves propagating in the air layer and dielectric A, respectively. Combining the propagation matrix of electromagnetic waves in each dielectric layer, the transmission matrix of the heterostructure can be obtained as:

$$M=D_{i\to A}{\left(p_{A}D_{A\to B}p_{B}D_{B\to A}\right)}^{N_{1}-1}p_{A}D_{A\to B}p_{B}D_{S\to B}{\left(p_{B}D_{B\to A}p_{A}D_{A\to B}\right)}^{N_{2}-1}p_{B}D_{B\to A}p_{A}D_{A\to o},$$ (5)

Thus, the reflection coefficient of can be expressed by $r=\frac{M_{21}}{M_{11}}$ and the reflectivity $R={\left|r\right|}^{2}$ is obtained. Although the absorption effects of the sensing layer may influence sensitivity, we have strategically excluded their quantitative impact on sensing perfor-mance from the theoretical model to streamline complexity and prioritize the investiga-tion of core sensing mechanisms.

Sensitivity, as a critical performance metric in biosensing systems, fundamentally governs device optimization. Accordingly, the sensitivity of the proposed structure is operationally defined as:

$$S=\frac{\Delta \theta }{\Delta n_{S}},$$ (6)

where Δθ denotes the resonant angle shift and $\Delta n_{S}$ represents the variation in the re-fractive index of HSA.

## 3 Results and discussion

**FIGURE 2:**
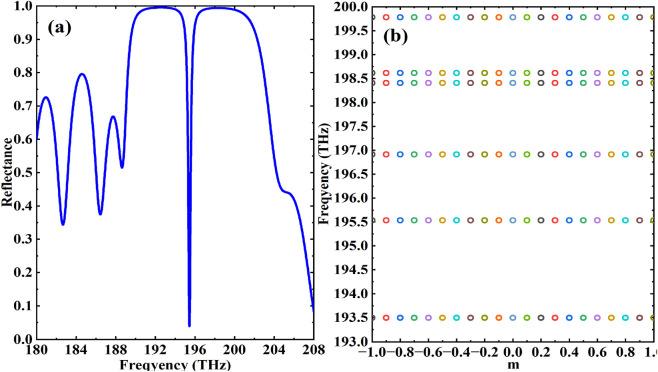
**(a)** The Reflectance spectra of the “PhC1 + PhC2” heterostructure; **(b)** The energy band of PhC heterostructure.

Conventional biosensing platforms, including surface plasmon resonance (SPR) and Bloch surface wave biosensors, universally rely on monitoring resonance peak shifts to detect minute variations in sensing layer properties such as refractive index. Adhering to this fundamental principle, our study first systematically investigates the angular-resolved reflectance characteristics of the proposed heterostructure. Numerical analysis reveals that although the direct stacking of photonic crystal 1 and 2 presents no distinctive structural features, the integrated photonic crystal heterostructure exhibits anomalous reflection characteristics with a sharply defined dip at 195.5 THz in its reflectance spectrum (Figure 2a). Subsequent band structure calculations further demonstrate the emergence of a characteristic TES mode within the heterostructure (Figure 2b). Crucially, the TES-induced sharp reflectance dip exhibits significant amplification effects on dielectric perturbations in the sensing layer, thereby establishing the essential physical foundation for achieving high-sensitivity HSA detection. Although a full numerical analysis of robustness against structural disorder is beyond the scope of this initial study, the inherent topological protection of TES suggests strong potential resilience to certain imperfections. A quantitative investigation of this property, e.g., by introducing controlled geometric disorders such as random thickness variations in the photonic crystal layers, represents a key focus of our planned future work to further validate the practical advantage of this platform.

**FIGURE 3:**
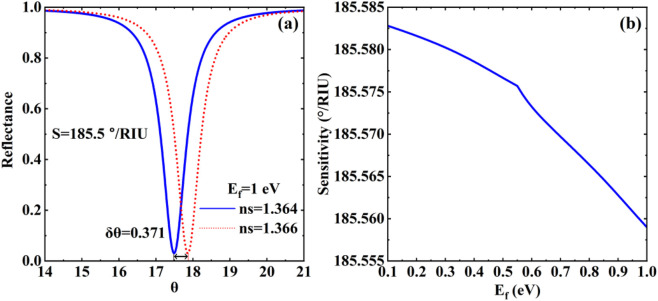
**(a)** The reflectance of the biosensor structure with respect to the refractive index of the different sensing layer at $E_{f}=1\text{ eV}$ , the reflectance of the biosensor structure with respect to the refractive index of the different sensing layer; **(b)** The sensitivity curve of biosensor structure relative to the Fermi energy).

In optical biosensing systems, four principal methodologies exist for sensitivity quantification: angular modulation, wavelength modulation, intensity modulation, and phase modulation. In this paper, since the resonance angle of the structure is very sensitive to the variation of the refractive index of the sensing medium, we are studying it based on an angle-modulated sensor. Based on the characteristic refractive index profile of HSA, we set the refractive index variation $\Delta n_{s}=0.002$ , with specific values $n_{s}=1.364$ and $n_{s}=1.366$ . The relaxation time τ was fixed at 1 ps with graphene layer number N = 1. Numerical simulations in Figure 3a demonstrate that topological edge state excitation in-duces step-function reflectance response at 195.5 THz, where reflectance plummets from baseline 1 to 0.05, creating a 95%-depth reflection dip. Quantitative analysis of the corre-sponding 0.371° angular shift yields a sensitivity of 185.5 °/RIU . Crucially, graphene’s Dirac cone electronic structure endows it with enhanced biomolecular adsorption stability compared to conventional metals. Its surface conductivity, dynamically tunable via Fermi level modulation through gate voltage, introduces new dimensions for performance opti-mization. In Figure 3b, the curves of reflectance *versus* angle of incidence for different Fermi energies are shown. The results show that the sensitivity of the sensor gradually decreases from 185.582 °/RIU to 185.558 °/RIU as the Fermi energy level increases from 0.1 eV to 1 eV . Based on the above findings, in order to further improve the sensitivity and ex-tend the measurement range of the sensor, we will discuss the influencing factors of the sensing performance in terms of the main parameters of graphene, the thickness of the sensing medium and the refractive index. Beyond HSA detection, the proposed platform holds significant promise for integrated diagnostic and therapeutic (theranostic) applications, particularly in conjunction with nanozymes. The robust field confinement of the TES could be utilized to enhance the local excitation efficiency of nanozyme-based photoacoustic contrast agents, thereby improving imaging sensitivity. Furthermore, the real-time, label-free refractive index sensing capability could be employed to monitor the catalytic process of nanozymes by detecting subtle changes in the local environment during substrate conversion. For instance, the adsorption of reaction products or the conformational changes of nanozymes upon interaction with targets could induce detectable resonance shifts. The electrical tunability of graphene further allows for dynamic optimization of the sensing condition to match different catalytic stages, potentially enabling closed-loop control of nanozyme activity.

**FIGURE 4:**
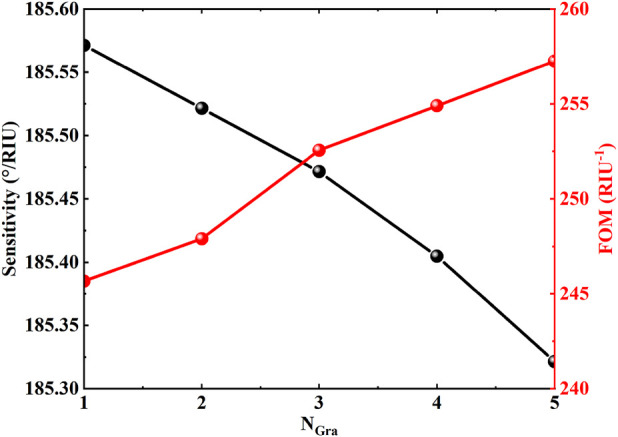
Effects of different graphene layers on the sensitivity of biosensor. Other parameters are the same as in Figure 2.

In Figure 4, the regulation of the sensor performance parameters by the number of graphene layers has been systematically revealed. When the number of graphene layers in-creases from 1 to 5, the sensitivity shows a monotonically decreasing trend from the initial value of 185.57 °/RIU to 185.33 °/RIU , which is attributed to the dispersion of the interfacial field distribution due to the stacking of multiple layers of graphene, which weakened the response of TES to the changes in the dielectric environment. In contrast, the quality factor FOM shows a significant upward trend with the increase of the number of layers. The FOM can be expressed as: FOM=S·DA ,and the quality factor DA is defined as DA=1/FWHM ,where FWHM is the full width at half height. The FOM increases from $245.1\;RIU^{-1}$ to $257.5\;RIU^{-1}$ when the number of graphene layers is incremented. This property is attributed to the broadening of the FWHM of the reflective dip with the increase in the number of layers, and although the sensitivity decreases, the FOM, which is the ratio of the sensitivity to the FWHM, increases due to a more significant increase in the de-nominator. Theoretical analysis shows that the FOM peaks at 5 layers even though the sensitivity decreases to the lowest value, reflecting the typical trade-off relationship be-tween sensitivity and resolution. The results also indicate that an excessive increase in the number of layers may lead to a broadening of the reflectivity curve, making it more difficult to accurately measure the resonance position under experimental conditions, and a balance between performance optimization and practical measurability needs to be sought. Collectively, these parametric studies suggest that for optimal sensitivity, the sensor should be configured with fewer graphene layers (e.g., 1-3 layers), a thinner sensing layer, and operated at a lower Fermi energy. A balance must be struck for specific applications, where a moderate number of graphene layers (e.g., 2–3) might offer a favorable compromise between sensitivity and FOM.

**FIGURE 5:**
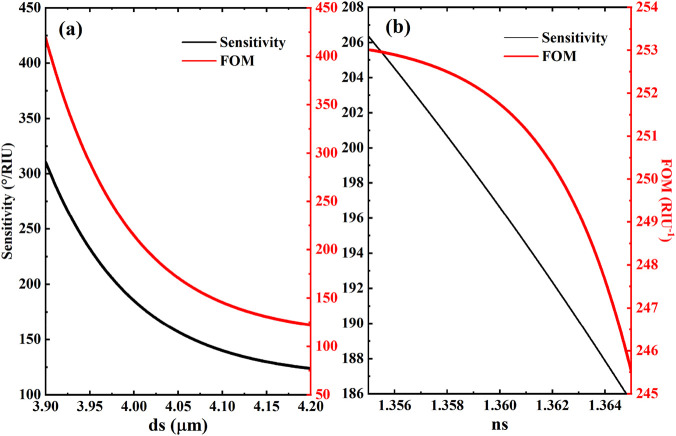
Effects of **(a)** the thickness and **(b)** the refractive index of the sensing layer on the sensitivity of biosensor. Other parameters are the same as in Figure 2.

The sensing layer thickness (ds), as a critical structural parameter governing system sensitivity, requires meticulous optimization for optimal detection performance. We systematically investigated the thickness-dependent sensitivity evolution through parametric analysis (Figure 5a). Theoretical modeling reveals a pronounced inverse correlation between ds and detection sensitivity within specific thresholds: both sensitivity and figure of merit (FOM) exhibit systematic degradation with increasing ds. This phenomenon originates from the TES mode’s ultrasensitive response to interfacial dielectric environments-excessive thickness compromises spatial field localization, thereby diminishing sensing capability. It should be noted that the calculated results are subject to some fluctuations due to multi-parameter coupling, and the final sensitivity and FOM values are obtained by calibrating the fitting algorithm. To elucidate the refractive index (ns) modulation mechanism, we established a quantitative sensitivity-ns correlation model (Figure 5b). Leveraging the TES mode’s field enhancement effect, minute analyte (HSA) refractive index variations induce substantial resonance dip shifts, resulting in linearly decreasing sensitivity profiles. Comprehensive analysis confirms that ns and ds jointly regulate pho-tonic local density of states through synergistic effects, collectively determining the sensor’s overall performance. These findings provide essential theoretical guidance for multi-parameter optimization in high-performance optical biosensor design. This integrated approach opens new possibilities for miniaturized, tunable, and interference-resistant biosensing systems… The topological protection mechanism of the edge state is anticipated to further enhance operational stability in complex media. For specific detection of HSA in real samples like serum, the graphene surface can be functionalized with selective capture probes (e.g., antibodies), while the robust TES core ensures a stable and sensitive transducer signal. Future experimental work will focus on such bio-interface engineering and validation using complex biological fluids.

## 4 Conclusion

In this study, we propose a novel optical biosensor for human serum albumin. The sensor combines the robustness of TES with the dynamic tunability of graphene’s electrical properties. Due to the excitation of TES, the reflectance exhibits a significant and sharp de-crease at 195.5 THz, which endows the sensor with an ultrasensitive detection capability of the refractive index change of the sensing layer, whereas the anti-jamming property of TES ensures a stable detection performance. The tunable conductivity of graphene intro-duces adjustable sensing characteristics. This work validates the feasibility of integrating topological photonics with two-dimensional materials for biosensing. The synergistic effects of TES and graphene enable simultaneous enhancement of sensitivity and dynamic tunability, opening new possibilities for next-generation optical sensing technologies in clinical diagnostics monitoring. This research validates the successful integration of topological photonics with two-dimensional materials for advanced biosensing applications. While this study presents a theoretical and numerical investigation, the experimental realization of the proposed biosensor is envisioned as the next critical step. Key challenges include the precise transfer and patterning of monolayer graphene onto the photonic crystal heterostructure, and the fabrication of defect-free photonic crystals with sub-nanometer precision. Advanced techniques such as chemical vapor deposition with PMMA-assisted transfer for graphene and electron-beam lithography for photonic crystals could be leveraged. Future work will focus on overcoming these fabrication challenges and experimentally validating the sensor’s performance. This platform demonstrates great potential in the application of treatment and diagnosis based on nanoenzymes. Its unique TES field confinement property can enhance imaging sensitivity, while its graphene tunable property enables precise control of catalytic activity, effectively addressing the key challenges of biocompatibility and performance consistency in current nanoenzyme technology. This work establishes a foundation for developing miniaturized, tunable biosensing platforms with broad prospects in clinical diagnostics, environmental monitoring, and personalized medicine applications.

## Data Availability

The original contributions presented in the study are included in the article/supplementary material, further inquiries can be directed to the corresponding authors.
